# A qualitative study understanding immigrant Latinas, violence, and available mental health care

**DOI:** 10.1016/j.dialog.2023.100112

**Published:** 2023-02-09

**Authors:** Tara Rava Zolnikov, Jose Luis Guerra, Frances Furio, Jessica Dennis, Carolyn Ortega

**Affiliations:** a California Southern University, School of Behavioral Sciences, Costa Mesa, CA, United States of America; b National University, Department of Community Health, San Diego, CA, United States of America; c California State University, Sacramento, Division of Social Work, Sacramento, CA, United States of America

**Keywords:** Latinx, Violence, Mental health, Domestic violence, Health, Women, Gender

## Abstract

Women from Latin American countries experience high levels of psychological and physical abuse and violence. Immigrant Latina women are often subjected to a patriarchal system in both family and government, which has resulted in a variety of complex and far-reaching outcomes. This qualitative study sought to understand the experiences of immigrant Latina women who were exposed to violence, as well as their access to mental health care. This study used 20 interviews with immigrant Latina women from El Salvador, Guatemala, and Mexico who had accessed mental health services in California. The primary themes that emerged from analysis of the data included motivating factors for seeking services (e.g., motherhood, community, hope, and mental health needs), barriers to accessing services (e.g., fatalism, marianismo, stigma, finances, language barriers, threats, abuse, and systemic insensitivity), and treatment and solutions (e.g., empathy, advocacy, and community approaches). These results appeared to be indicative of the importance of addressing sociopolitical, historical, and cultural trauma as an imperative component of effective treatment. In this context, the authors explore liberation psychology, a concept and approach that promotes social justice values and emphasizes the empowerment of immigrant Latina women in clinical practice. It is recommended that the historical sociocultural abuse of immigrant Latina women be thoughtfully considered and discussed in the therapeutic process to create lasting psychological change. Future research, policy efforts, and program development, including psychotherapeutic treatment modalities, should focus specifically on marginalized groups facing barriers to mental health care in order to increase access and effectiveness of treatment.

## 1 Introduction

The National Coalition Against Domestic Violence (NCADV) defined intimate partner violence as willful intimidation, psychological and emotional abuse, physical and sexual assault, or abuse perpetrated by one partner against the other [1,2]. The psychological and interpersonal implications resulting from intimate partner violence has been researched for many years. Researchers have found that Latina/o partnerships reported higher rates of intimate partner violence (14%) than White couples (6%) with re-occurrence rates of 59% among Latina/o couples and 37% among White couples [3]. In fact, about 23.4% of Latina females in their lifetime and 48% of Latinas reported that intimate partner violence increased after they immigrated to the United States [4].

Women have historically been oppressed by society's unspoken rules and abused by men in many patriarchal communities [5]. Unfortunately, for immigrant Latina women, living in a new country with or without legal standing has made it difficult for those in abusive intimate relationships to seek assistance in a society that has not recognized their existence [6,7]. Immigrant Latina women are in a unique position, especially if they suffer from intimate violent relationships with their partners, because women often remain in these relationships for reasons ranging from financial support and dependency, familismo, marianismo, machismo, verbal or physical threats, physical and sexual abuse, cultural shame, fatalism, and feelings of guilt [8]. Indirectly, cultural disparities and a lack of autonomy have produced an adverse psychological effect, making women believe they are alone and do not have a voice [9,10].

Fatalism and familism may further compound this issue. Fatalism, a distinctive characteristic of Latino culture, can be described as a strong belief that problems, including violence, are determined by fate and destiny [8]. Further, familism has underscored women's sense of obligation to their children as a strong motivating factor for remaining in abusive relationships and sacrificing their own well-being [8,11]. As a result, women who have immigrated from Latin America regions may have been socialized to be docile and accept partner violence as part of everyday life and their commitment to their marriage [8]. Immigrant Latina women have encountered additional unique obstacles, such as traditional and cultural gender roles, isolation from families of origin, fear of deportation if they decide to leave their husbands, and the difficult navigation of oppressive systems [7,9]. To compound these difficulties, immigrant Latina women who experience domestic violence may also likely suffer from other types of abuse, including financial, sexual, physical, and psychological abuse involving children and immigration status privilege [12]. The socialization of traditional gender roles has successfully reinforced the social constructs of machismo and marianismo, reinforcing the constructs that have formed these destructive influences [8].

Immigrant Latina women may be conditioned to believe that the solutions to these problems must come from external sources, such as their husbands or the government [13]. Many of these issues have perpetuated the cycle of violence and sexism normalized by systemic inequality and ongoing cultural trauma on certain subgroups [9,13]. Ironically, some laws that have been passed to protect people in California have actually criminalized immigrants and normalized violence perpetuated against immigrant Latinas [10,14,15]. Enchautegui and Menjívar [10] defined *legal violence* as the indirect effects of politics, laws, and regulations which have further alienated women from seeking assistance. For example, the United States immigration court system marginalizes women who decide to seek legal assistance by implementing punitive laws and policies against them [9,14,15]. Secondly, the child welfare system has implicated and criminalized immigrant Latina women by creating high demands when the women have lacked the financial resources to comply, ultimately leading to labeling of these women as ‘bad’ mothers [16,17]. Earner [16] stated the child welfare system have barriers that make the immigrant mothers harder to get services such as restrictions due to the family unit still connected but most of the services are for single mothers. There has been research on the government services immigrants receive to help assess the demand, however, due to the restrictions there is limited information [16,17]. The federal systems have limitations on services based on citizenship which cause immigrants to have a harder time to access services [17]. This type of legal violence leaves women unprotected, vulnerable to abuse, and without any options for change [14].

Therefore, one cannot fully conceptualize the effects of violence against immigrant Latina women without first understanding the unwanted conditioning that is imposed on them in their birth countries [5,18]. After all, domestic violence has been a result of a number of psychosocial factors, such as the political environment, which has invalidated women's rights. Further, fatalism has caused women to believe abuse might be ‘part of their destiny,’ while cultural values driven by religious doctrines have subjugated women as less than and submissive to their husbands, ultimately promoting machismo and marianismo in a society which has promoted inequality [5,10]. Through the process of social conditioning and reinforcement, both women and men have maintained the prescribed gender roles, normalizing violence against immigrant Latina women [10].

Thus, the purpose of this study was to identify the obstacles that have prevented immigrant Latina women from seeking mental health treatment, the motivating factors for seeking help, and ways in which mental health treatment might be tailored using a liberation psychology framework. The expectations of procuring this information were to highlight the mental health needs of immigrant Latina women while considering both past traumas in the native country and traumas experienced pot-migration. We also aimed to identify effective treatments approaches.

## 2 Methods

This qualitative study used an interpretative phenomenological approach as a research tool and strategy to explore the lived experiences of immigrant Latina women who were exposed to violence, as well as their access to mental health care. This method and approach was utilized in an effort to understand these women's experiences, highlight their unique perspectives [19,20], and allow for examination of each individual's current experience, as well as their world and personal constructs based on past experiences [21,22]. The phenomenological approach also assumes that the researcher's perceptions and preconceived ideas are set aside, enabling them to enter into a participant's subjective experiences [20].

The American Psychological Association [21,22] determined that the use of qualitative methods was more sensitive to personal subjectivities and social complexities than methods which have relied on pre-existing frameworks. The use of qualitative methods—and more specifically, a phenomenological study—allowed the researcher to ask open-ended questions to collect information on participants' cultural differences and meanings [23]. Because of the unique complexities of Latin American cultures, this study used open-ended inquiry to understand differences across cultures (e.g., sociopolitical experiences, language, and cultural norms) [21,22].

The theoretical foundation supporting this research was liberation psychology, which is an approach that seeks to liberate the oppressed [women] through self-empowerment and understanding of their narratives while re-creating their existence in a macropolitical context of politics, religion, and economic and gender dynamics [5]. We utilized liberation psychology as a theoretical framework in this context due to its promotion of social justice values and emphasis on the empowerment of immigrant Latina women in clinical practice. It was in this way that we aimed to thoughtfully explore historical sociocultural abuse of immigrant Latina women and the importance of considering these in the therapeutic process to create lasting psychological change.

### 2.1 Data collection and analysis

This study used 20 interviews with immigrant Latina women from El Salvador, Guatemala, and Mexico who had accessed mental health services in California. Interviews were conducted with immigrant Latina women who had migrated to the United States, were at least 18 years old, and sought mental health services. Researchers set out to recruit participants from Latin American countries with the highest rates of violence toward women, including Mexico, El Salvador, Honduras, Nicaragua, or Guatemala. Participants recruited were from Mexico, El Salvador, Guatemala, and Colombia [24,25]. That said, it is important to highlight that while participants were from Latin American countries and culture and language may be similar, differences do exist based on geographical regions. This research project received IRB and ethics approval from California Southern University.

Convenience sampling was used to recruit volunteers for this study. Participants in this research were all located and receiving mental health services at a practice within the San Fernando Valley region of California. The San Fernando Valley region of 436.7 mile^2^ has a population of 1,856,209 and is a subdivision of Los Angeles County (U.S. [26]). Within this population, 43% identified as Latinx, and 40% of the population had annual household incomes less than $50,000, with 15.2% below the poverty line. Moreover, 48% were foreign born from Latin America [26,27]. Participants were referred to the practice through California's Victims of Crime program. All had experienced violent crimes [domestic violence] while living in the United States. Although the participants were referred to the group private practice by the program, survivors had the freedom to choose any provider in the state.

Creswell [23] indicated that for a qualitative phenomenological study, three to ten participants were preferred or until saturation occurred. This study aimed to recruit at minimum 10 to 20 participants. The face-to-face interview was expected to last 30 to 45 min, depending on the participant's openness and willingness to share their experiences after being prompted by open-ended questions. In total, the researcher carried out 20 interviews before reaching the saturation point.

Prior to beginning the face-to-face interviews and audio recordings, the investigator reviewed the informed consent with the participant. No compensation was provided to participants. Pre-formatted, open-ended questions guided the collaborative part of the individual interviews, which included the following major themes: (a) the participant's background such as the reasons for immigrating to the United States, (b) the participant's interpersonal experiences including transitioning and adjusting to the United States, the availability of support and resources, and experience as an immigrant Latina woman living in the United States as compared to living in her country of origin, (c) accessibility of services such as the motivating factors in seeking mental health care, and reasons that the participant initiated seeking mental health care.

All interviews were recorded using a standard Sony audio voice digital recorder. After the interview recordings were transcribed and deidentified, the data was analyzed via hand-coding and further “winnowed” ([23], p. 195). Winnowing is the process by which some of the data is disregarded while other data remains as the focus of the analysis [23]. For the purpose of data analysis and coding, a heuristic conceptual framework was used, as described by Moustakas [28]. This process employed bracketing to suspend presuppositions and reducing and eliminating unnecessary information that did not provide an understanding of the research phenomena (e.g., redundancies). All data elements from transcribed information was of equal value. Data was then clustered, viewed, and analyzed for emerging themes [23,28,29]. A codebook was then developed, which had approximately 15 codes (e.g., “motherhood” and “stigma”). From the codebook, the researcher organized quotes and content among the themes and sub-themes, which represented information to support the participants' lived experiences, including the motivating factors for seeking mental health care, barriers preventing access to this care, and the re-examination of potential treatments and solutions.

### 2.2 Validity

The purpose of using a phenomenological study was to understand the multiple realities, perceptions, perspectives, and experiences to highlight rich and authentic knowledge of the phenomena [[30], [31], [32]]. In phenomenological research, the validity and reliability of the research was based on the credibility, trustworthiness, and authenticity of the information collected during the course of this study [23]. A phenomenological design collected subjective information provided by the participants which required careful consideration of the validity and reliability of the protocols utilized [31]. To ensure the rigor of data collected, the researcher considered reliability and validity of the qualitative data as part of the data analysis process. Validity and trustworthiness were furthered increased by making explicit biases and acknowledging subjective judgements [23,29].

Validity was also achieved through low inference descriptors extracting from participants verbatim [29]. Reliability of the data included disclosure of personal orientation and context and ensuring accuracy in recording and transcribing, since most data was translated from Spanish to English prior to data analysis [23,29]. Peer debriefing and auditing were also used to further address the issues of validity, reliability, credibility, trustworthiness, and authenticity. Once the data was transcribed, it was reviewed by another research peer to ensure that personal biases based on the researcher experiences did not influence the data. The study protocol and ethics review were approved by the university's institutional review board. All participants provided informed consent prior to the commencement of the interviews and audio recording, and codes were immediately assigned to every participant to ensure deidentification of the data.

## 3 Results

Throughout analysis of the data, several primary themes emerged. These primary themes included motivating factors for seeking services (e.g., motherhood, community, hope, and mental health needs), barriers to accessing services (e.g., fatalism, marianismo, stigma, finances, language barriers, threats, abuse, and systemic insensitivity), and treatment and solutions (e.g., empathy, advocacy, and community approaches). Liberation psychology, an approach that aims to liberate and empower oppressed folks through the promotion of social justice, was utilized as a theoretical framework for this qualitative research study. While engaging in the research and data analysis process, we aimed to not only explore the historical and sociocultural experiences of immigrant Latina women, but also how consideration of these may be beneficial in the context of clinical practice and the therapeutic process.

### 3.1 Demographics

The participants in this study resided in the northeast San Fernando Valley area located in Los Angeles County, California. Of the participants, three were single, one was widowed, two were divorced, nine were married, and five were in cohabiting relationships. A total of 16 women reported to have one or more children, while four did not have children. Five of the participants were employed and 15 were unemployed. There were 12 participants that identified as bilingual (English and Spanish); six as monolingual Spanish; and two who reported speaking Quiché (also spelled K’iche’) secondary to Spanish they learned while in the United States. Quiché is a Mayan language and the second language (after Spanish) spoken in Guatemala. Of the 20 participants, 11 were from Mexico, six were from Guatemala, one was from Colombia, and 2 two were from El Salvador. The ages of the participants ranged from 27 to 58 years old. The length of time participants had lived in the United States ranged from five years to 40 years. Participants reported that they had been from three to 23 years of age when they arrived in the United States. The data spoke to the level of acculturation and the years of possible pre-exposure to traumatic experiences living in Latin America. This allowed comparison between recent arrivals and those who had experienced most of their lives in the United States.

### 3.2 Motivating factors for seeking services

The data provided information on some of the participants' main motivators to seek mental health care, even in the face of challenges and culturally rooted norms and beliefs related to self-sacrifice and pieties. The primary motivating factors reported by the immigrant Latina women in this study included the role of motherhood, a sense of community, ongoing hope and optimism about the future, and a variety of mental health related needs. Overall, these women demonstrated resiliency, as evidenced by their continuous efforts and belief that life could be improved.

#### 3.2.1 Motherhood

A common motivating factor to seek mental health care reported by the women in this study involved the role of motherhood and concern for the well-being of children. These women had been socialized and conditioned to prioritize the needs of their children; otherwise they would be judged as selfish or bad mothers. Women learned to keep their mental health related struggles to themselves, which often involved ignoring their emotional and psychological needs. However, motherhood served as a blessing for many immigrant mothers because it had encouraged them to seek outside help and maintain hope. Participant M004 stated how she sought mental health care:

The reasons that helped me or that led me to seek therapy was because I have a son who was a little bad [he had a drug use problem], and all that led me to seek help. And it helped me a lot as much as it helped my son. For me it was a very good and very beautiful experience.

Participant M0018 indicated that as a child she was sexually molested but had never sought any type of counseling services. She also reported experiences of domestic violence in her last relationship with her ex-husband while living in the United States. It was not until the suggestion of a healthcare provider that she enrolled her son in mental health services because of the domestic violence. It was only then that she decided to utilize counseling services.

#### 3.2.2 Community

The theme of seeking a sense of community was underscored as an important factor in these women's initial decision to seek mental health care. Participants indicated they had heard other women who were neighbors talk about their experiences. The normalization of seeking outside help by older women in their community had empowered them to first consider the possibility of mental health care and then to seek mental health care. Participant M0014 reported:

Well, I am one of those people who likes to listen to older people share their stories, [they would say] that they had their therapists [to talk or to “vent” about problems], and they [the older women in the community] explained to me that it [mental health care] is not for crazy people.

The role of community centers such as healthcare facilities had encouraged participants to consider mental health care. It seemed that the participants had been motivated to seek mental health care when they were encouraged by their primary care physicians or other people in the community. It helped that those in the community spoke Spanish. Often, the women learned of these centers from their neighbor's recommendations, and then got to know each other better in these centers. Through these experiences, women were able to hear each other's stories, have their struggles validated and normalized, and become comfortable with sharing their experiences outside of the family. This process further contributed to the forming of their own sense of community.

#### 3.2.3 Hope

Participants indicated that living in the United States gave them a sense of hope for a better life and appreciation for their lives as women. Participant M0010 said,

I was working like crazy, 63 h a week, driving, you know like that was my work, I was the only financial support in the home, he wasn't working, he was doing absolutely nothing and then I said this is it, I don't want this life [for me and my children], I don't like this life, I don't like [it] that I am getting really, really tired.

Participant M0010 explained the many challenges she had encountered, as well as her commitment to not giving up and her dedication to regaining custody or her children and making a better life:

I became homeless, fortunately I kept my work, I was working, couch surfing, and was able to pay for my programs, and finish my programs, eventually I recovered my kids and then after I recover my kids I was able to enroll in therapy and actually go to therapy.

#### 3.2.4 Mental health needs

Participants reported that once they had gotten to their breaking points and had realized there was something wrong with their mental health, they were more likely to get care for themselves and for their children. The previously described hope provided these women with motivation, as well as the space to reflect on the help they needed to reach their goals. Participant M005 shared:

I went to the doctor and as I was sitting, I was bawling non-stop and he [medical doctor] told me I had postpartum depression. She [the doctor] actually mentioned therapy to me. And she suggested that I call the mental hotline and start my referral.

In summary, the interviews with immigrant Latina women attested to the fact that the role of motherhood and a sense of community played a significant role in the pursuit of mental health care. They indicated that they were becoming aware of their own mental health needs but did not act on seeking the services until they had been encouraged by others in the community, such as elders or medical professionals. Throughout these experiences, participants explained their efforts to maintain hope and commitment to themselves and their families.

### 3.3 Barriers to accessing services

#### 3.3.1 Fatalism, marianismo, and stigma

Participants reported that one of the barriers to seeking outside help for themselves had been the belief that it was part of a woman's destiny to accept the experiences lived in her marriage. Participant M002 stated, “first of all, culture, we [women] don't talk about it [mental health, relationship problems] and it is discouraged to talk about outside of the family.” Researchers have referred to this phenomena as fatalism, a philosophical belief that women should subjugate situations and lived experiences by accepting their destiny which has been predetermined [8]. Participant M001 explained, “it is like women are nothing over there [Mexico], the role of a woman is to their children and [to] raise them.” Additionally, participants referred to themselves as ignorant and referenced other stigmas related to mental health care. For example, the use of the word *crazy* was mentioned often during the interviews: “the comments that my family made is [that] only crazy people go to therapy,” stated PM001.

A deep-rooted belief associated with Christianity within Latino culture is the concept of marianismo, which asserts the belief that women needed to maintain their roles as wives (pure and morally strong) and mothers (self-sacrificing) and prioritize the well-being of their husbands and children [[33], [34], [35]]. The sense of guilt in some of the participants rendered them incapable of daring to take any action that may contradict submissiveness and self-sacrifice. Participant M0010 stated that she was criticized by both her ex-husband and mother-in-law, the latter stating, “You are not a humble woman, you do not respect your man—your husband.”

A sense of helplessness was a common theme among most of the women. Fatalism prevented many of the women from considering solutions or alternative possibilities and instead led them to feel they needed to accept their experiences as a normal part of life. One of the participants, M0013 explained, “You don't talk about your problems, you just suck it up, and you pray, and you move on.” Another woman indicated that her ex-husband would often use the Bible to justify his abuse toward her as a woman and a wife which made it difficult for her to justify a reason for herself to seek therapeutic services. Participant M001 stated that in addition to her family of origin telling her that counseling was only for crazy people, she was told, “Maybe you just need God.”

Participant M0010 shared her experience when her ex-husband used religion to justify the submissiveness of her as a woman, a wife, and a mother. She stated, “He used to use the Bible to validate his actions [like hit me, isolate me from my family] and the way he wanted, his control and power, that's law and you have to obey that's what it is.” Another woman (M0020) explained her experience when she finally decided to seek outside help by going through Christian counseling. She indicated that the clergy discouraged her from leaving the relationship and getting a divorce as it was against the laws of God.

#### 3.3.2 Finances

Financial barriers were reported by all of the participants in relation to difficulty accessing mental health care. Participants reported that they had not had the financial resources to seek counseling or therapy services. Some of the women who were employed indicated that their ex-husbands managed their money even if their ex-husbands did not work. Participant M0016 described her experience:

I worked and [my ex-husband] would take all the money from me, he would wait for the week to end, and he would buy his beer and kept the rest of the money, I didn't even get one dollar for me and then he would beat me, hit me in my stomach.

Another participant, M002, described the limitations that she had encountered reducing the ability for her to access treatment: “affordability, because if we recently immigrated and our access to anything is limited because of legal status and if it is available it is very little and limited.” Participants also discussed their experiences being socialized to fulfill gender role expectations as a woman and being denied the opportunity to seek employment. Participant M004 stated, “In my country, as a woman my duty was to stay home and do housekeeping; we were very poor, but that did not matter, there was no work for women.”

#### 3.3.3 Language barriers

Language barriers when they immigrated to the United States were reported by all of the participants. The majority of the women had experienced a difficult transition when adapting to the American culture, and this was only made worse because of language barriers. Participant M001 described the challenges she had faced while acculturating to the American culture because of the lack of proficiency in the English language, “I didn't learn another language, so I had a hard time learning the language, it was hard.” Another participant M0016 provided her experience:

It was very difficult for me, I did not speak Spanish or English, I spoke Quiché, there were times that I didn't eat because I could not speak the language, I would get lost if I went out, and I did not know how to read. [My ex-husband] brought me to the United States with him [from Guatemala] and then he started to beat me a lot, I would bleed a lot, and he [ex-husband] took advantage of me because I couldn't speak the language.

#### 3.3.4 Threats and abuse

A number of women indicated an obstacle to seeking mental health care had been the fear of losing their children after threats were made by their ex-husbands. When female participants had attempted to empower themselves and work toward improvement of their lives, the threat of not seeing their children again had been enough to render the women helpless and hopeless. Participant M0017 stated that her ex-husband had threated to kill her numerous times if she contacted the police or if she wanted to leave the relationship:

In the house he [ex–husband] always kept a knife, it was like a little sword hung on the wall next, and he told me that [knife] was meant especially for me. He grabbed the knife again and went to where I was, I sat on [the chair by the kitchen] and he put the knife (points to side of stomach). He then told me to put my son to sleep or “I kill you in front of him.”

Another major theme was the physical and psychological harm caused to the women by their partners. One of the women had endured 11 years of physical and psychological abuse due to the threat of being killed by her husband and never seeing her children again. For some of these women, the barrier broke when their children had decided to contact law enforcement when the women themselves would not have called the police.

#### 3.3.5 Systemic insensitivity

Participants felt that the social services system and the legal system blamed the mothers and favored the ex-husbands. A participant shared her disappointment with the social services system because her children had been removed from her care by the social worker. Participant M0010 described her experience in which the Department of Children and Family Services had been unhelpful and had added financial and emotional stress. She described the number of barriers she had encountered in satisfying the requirements of that department, making it difficult for her to seek her own mental health care because she was functioning in survival mode. Participant M0010 reported that when she tried to reach out to providers from the list given to her by the department, “I had to pay for all the services, honestly I couldn't make it; no support.” It was not until a year later that participant M0010 was able to enroll in her own mental health care.

The barriers and obstacles that prevented these immigrant Latina women from seeking mental health care were experienced both internally and externally. Marianismo, machismo and fatalism were common when reporting the obstacles that kept women from seeking mental health care. Unfortunately, when women decided to seek outside assistance, many times governmental agencies became involved and further overwhelmed or discouraged them.

The data collected from the participants provided vital information about common barriers that immigrant Latina women faced that prevented or made it difficult for them to access mental health care. The primary barriers included those related to fatalism, marianismo, stigma, finances, language barriers, threats and abuse, and systemic insensitivity.

### 3.4 Treatment and solutions

This study aimed to inform current practices and serve as historical markers for future practitioners working with minority groups that had extensive historical traumas experienced in Latin America and experiences while living as immigrants in the United States. The statements made by the participants pointed to the important of empathy, advocacy, and community approaches in future efforts related to treatment and solutions. It is recommended that further examination can be given to address the root of the problems of patriarchal systems and misogyny rather than just treating the symptom of intimate partner violence.

#### 3.4.1 Empathy and advocacy

Participants indicated their decision to seek mental health care often had been made when they were at breaking points. In a sense, participants had sought treatment coming from a constant state of stress and emotional crisis. One of the participants (M0014) shared her experience with a social worker who had made her feel judged and shameful. In another case, a participant indicated that the most successful support had been felt from her psychologist by whom she felt understood and listened to. As practitioners, offering a voice as an advocate could greatly build on the trust with the practitioner and the likelihood of continuing with their mental health care.

Based on the participants' stories, the themes for successful treatment approaches focused on advocacy and having a voice to speak up for their needs. Current clinical approaches could increase effectiveness if case management and resources assistance was heavily emphasized when working with immigrant Latina women. This is because many of the women's first outside experiences had been with mental health providers with whom they had built a therapeutic relationship increasing the likelihood of them using the resources that had been provided to them as part of their treatment.

#### 3.4.2 Community approaches

Accepted treatment approaches have offered helpful interventions for patients seeking mental health care. More emphasis on case management during the treatment process was appreciated by the participants because it offered them a sense of relief, direction, and empowerment. Some of the skills that were helpful were problem solving strategies and assistance in providing the linkage to other programs and services. Participant M0020 stated that she was helped by the school to connect her children to counseling services and she felt supported by the school and the counseling center.

While individual therapy proved to be helpful for most of the participants, the inclusion of their children in treatment appeared to be important in their decisions to seek and continue mental health care. Participants indicated they would have not sought treatment for themselves but had done so only after they were able to ensure that the children would receive treatment as well. One of the participants (M0020) described feeling hesitant to engage in mental health care, but stated that she had done so as part of family therapy with her children.

Community-based solutions and action-oriented interventions offered concrete assistance valued by the participants. Providers could make efforts to increase the interrelationship with others in the community where the client lives. Additionally, the incorporation of family therapy seems to have been one of the best avenues to engage these immigrant Latina women to seek mental health care. This was because the women placed a priority for their children and families' well-being. Thus, making family therapy part of the treatment would respect the cultural aspect of family which could make Latina women feel understood and welcomed.

## 4 Discussion

In Latin America, the incidence of violence and abuse against women is higher compared to other parts of the world [[36], [37], [38], [39]]. Consequently of the increase in research, various treatment approaches have been developed to address the sequelae of intimate partner violence experiences during psychological treatment. Immigrant Latina women often do not seek assistance or mental health treatment when needed due to a variety of factors, such as cultural and linguistic barriers, ignorance of access to services, and mistrust of the legal system [8,16,40]. This calls for solutions specific to this population. Barrera and Longoria [8] noted that while there have been many efforts to increase cultural competency in the field of psychology, it has likely been insufficient because a traditional Eurocentric approach might not address the unique experiences and challenges of individuals. This approach may fail to recognize psychosocial stressors—financial, sociological, historical, environmental, and political—these women have experienced in their native countries [8,18]. While a solution to overarching change to large populations may be difficult to find, smaller, more obtainable solutions may be employed. Violence against women can have long-term adverse effects on the psychological development which can inhibit a productive and happy life. Nevertheless, therapy can help mitigate these effects and can offer women tools to build resilience.

The data collected in this study helped provide some insight into the lives of immigrant Latina women. Some of the common themes among the 20 participants were: marianismo, fatalism, financial limitations, language barriers, and different types of psychological and physical abuse, including sexual and physical abuse and verbal threats. The findings of this study attest to how important it is for mental health practitioners to be aware of the complexities of traumas experienced by immigrant Latina women living in the United States. Repeatedly, the participants indicated the sense of hopelessness and acceptance of their life experiences. Further, all of the participants described their initial experiences with helping professionals would be indicative of whether they would continue to seek treatment. Therefore, it is imperative to be cognizant of the deep-rooted acceptance of patriarchy, machismo, misogyny, and gender violence reinforced by the culture and religion in the field of psychology. Efforts to eradicate or circumvent the barriers that immigrant women encountered when seeking mental health care as well as available clinical approaches could be tailored to the needs of immigrant Latina women. For example, many Latina women have been socialized to accept fate and destiny by oppressive systems such as institutional sexism and misogyny [33,34]. Furthermore, intimate partner violence has been the result of a patriarchal hypermasculine system which produces the abuse (or symptoms) of women. The oppressive patriarchal systems in many Latin America countries have normalized inequity and injustices against women.

Historically, countries in Latin America have a reputation for having the highest rates of gender abuse toward women [36,37]. It has been documented that women have experienced high rates of psychological, physical, and emotional abuse, yet many of the women have failed to utilize available mental health care [36,37]. Previous research has been conducted on the issue of mental health and Latina women but there has been minimal research on immigrant Latina women. In order to address the mental health needs of immigrant Latina women, the need to identify effective treatments approaches is vital. These treatment options should not only address psychological symptoms, but deep-rooted cultural beliefs of fatalism, machismo, and marianismo concerning the role of women, and social and political realities experienced by immigrant Latina women to understand their stories and rationalization.

Latin America is very diverse and rich in culture. The women who participated in this research study were from the Latin America countries of Mexico, Guatemala, El Salvador, and Colombia. Although they had immigrated from various countries and different geographical areas, the systemic oppression and institutional sexism experienced by these women appeared to be globally distributed. The common themes concerning the motivation to seek mental health care encapsulated within the interview responses included the role of motherhood, the role of the community, the concept of hope, and the need for mental health care.

On the other hand, the common themes of factors that prevented these immigrant Latina women from accessing and seeking outside assistance such as mental health care were the belief of fatalism, religion and marianismo, mental health stigma, financial limitations, language barriers, and the psychological, emotional, physical and sexual abuse and threats toward women. Lastly, the role that systematic oppression and patriarchy has played further perpetuated the normalization of the subjugation of immigrant women and the conditioning of their psyche. Participants described their experiences being judged by other women, or not having a place to share their story. The community advocacy and treatment options may be more utilized than individualized care due to the cultural implications; however, individual therapy has proven to be effective.

Consistent with other research studies, the participants in the study confirmed that immigrant Latina women had experienced cultural-historical trauma that had never been addressed, nor had they sought care for it themselves [8,9]. The concept of marianismo which was derived from Catholicism, has reinforced the female gender roles in Latino culture. Immigrant Latina women have been conditioned to accept traditional gender roles as women and have been expected to venerate moral strength and purity. Not surprisingly, many of the participants relied on religion as a source of emotional support. At the same time, religion has served as an institution to normalize violence and abuse of many women in Latin America.

Furthermore, the role of fatalism in the lives of the women has been influential in their decision-making and problem-solving, often impacting whether they had sought outside care such as mental health care. Many of the participants confirmed the role of fatalism in the lives of immigrant Latina women and the impact it had on their decision-making and rationalization, which has often been misunderstood by mainstream society. Also, participants expressed their resignation to life events and circumstances whether positive or negative and accepted these experiences as part of their fate. When issues arose such as relationship issues, motherhood, and the need for emotional and psychological support, participants reinforced the concept of fatalism as part of God's will and predetermined destinies.

### 4.1 Historical trauma and liberation psychology

It is crucial to help survivors by acknowledging and validating the impact of their past historical and cultural traumas within psychological treatment. In this context, decolonizing or decolonization is defined as a “process of examining and undoing unearned privilege” as a result of historical injustices on oppressed groups [41]. Thus, the importance of addressing the needs of immigrant Latina women in close abusive partnerships calls for attention to the issue of the subgroup of interest. Great strides have been made toward increasing social justice and cultural awareness in the psychological treatment of diverse groups of people, including Latina women and intimate violence [42]; though, there are shortcomings that continue to exist. Eurocentric approaches have failed to acknowledge these factors placing immigrant Latinas women at double risk, first at the hands of their partners and second with the involvement of the state deeming survivors as failed wives, mothers, and women [11,16].

Liberation theology has promoted commitment to oppressed people, the poor, and the marginalized as described in the Gospels of the Catholic Church and the Bible [5,13]. Thus, during the 1980s, liberation psychology was born in Central America within the context of oppression, violence, and poverty [5]. Martín-Baró resisted hedonism and universalist thought which focused on scientism and evidenced-based practices rather than on analysis of lived experiences [5,13]. Liberation psychology involved questioning the dynamics of psychology and the practices used to help people escape from power structures of oppression and achieve psychological freedom and liberation [5,13]. Liberation psychology has emphasized critical consciousness by “decoding the social lies that naturalize the status quo while searching for alternative interpretations of one's situation” ([13], p.18). Liberation psychology was designed to address not only psychological needs but also socio-political and cultural factors which impact women's ability to leave violent relationships [43]. Watkins and Shulman [13] explained that doing otherwise would erase women's historical memories and reinforce the belief that women should remain silent and that their experiences did not matter. An intervention for Latina women who experience domestic violence will need to be grounded in the specific experiences of this group and their social conditions (e.g., familism, ADD) to be able to offer the analysis, process, and practice of liberation psychology.

In specific, liberation psychology dovetails into the call by the American Psychological Association to practice from a multiculturally attuned and ethical framework as is affirmed within the professional code of ethics. That is, the status quo of graduate level training and overarching conventional practice in the field in providing psychotherapy remains focused on centering the delivery of services from a Eurocentric perspective where the patient must adjust to parameters that are not always flexible, or cognizant, of the patient's personalized circumstances [44]. For example, Gallardo and authors in the journal, *Professional Psychology: Research and Practice*, indicate that practitioners of the healing arts take for granted that factors inherent to the therapeutic encounter such as appointment times, travel distance to clinical settings, offices that do not reflect the cultural environment the patient may be comfortable with, interactions with other office staff, the expectation of divulging personal information with a provider of treatment that they yet have to form an alliance with, and even mode of delivery (i.e. use of worksheets, charts, and other psychotherapy within or between session tools), as elements that diverse patient populations are inherently socialized to expect in seeking behavioral health treatment.

However, culturally responsive treatment, such as informed by a liberation psychology framework, can help practitioners to understand their clientele by validating their intergroup diversity, even among other Latino populations. Latinos are not a monolithic population, with a variety of trajectories of the origin of their trauma, linguistic nuances, socio-political histories, economic resources, and religious diversity. As noted by Gallardo et al. [44], liberation psychology is indeed a culturally informed model that provides an avenue for behavioral health providers to both inform and mitigate perpetuating the psychotherapeutic dilemma of wielding a power differential over a patient, who is already traumatized. The APA Code of Ethics specifically opens its aspirational Principles with the guidance “to do no harm” (Principle A) as well as notes in Principle E that practitioners should respect the rights and dignity of individuals [21,22]. The use of liberation psychology provides a unique healing experience where the phenomenology of the patient is validated, integrated, and the patient is encouraged to live authentically within their cultural ecology.

### 4.2 Implications for professional practice

Based on the results from this study, we recommend that practitioners in the field of psychology working with immigrant Latina women should continue to be mindful not to become part of the oppressive structures (i.e., inequity of the power differential) that these women historically have experienced [45]. By working to ensure that immigrant women have a positive therapeutic relationship, practitioners could send a positive message about therapy to other women who might be reluctant to seek mental health care. It has been through word of mouth that many have learned about services available to them. The unique struggles of immigrant Latina women are intimately tied to their social, political, and historical culture and may require effective engagement to seek care. This is a significant point because oppressive social conditions have been direct results of psychological oppression of women of color since people are influenced by their social context.

The trauma inflicted on Latina women in Latin American countries by their partners as well as governmental institutions have made women fearful and cautious of seeking out mental health care [8,16,40,46], and it has perpetuated the cycle of violence and of silence on women. Thus, increasing sociopolitical consciousness, women could become more conscious of themselves and their lives [45]. The conscious awareness could motivate women to recognize the realities of their oppressive situations. Additionally, liberation psychology has contended that through the process of de-ideologizing reality, women could be encouraged to break away from ideologies that have been developed by those in power and instead to promote their ideologies based on their lived experiences and their realities [45]. By endorsing their own ideologies, immigrant women can be encouraged to develop their own interests and needs for their well-being.

Several possible solutions could be implemented to increase awareness of mental health services, change the mental health stigma, and improve accessibility for immigrant Latina women. There is a great need to increase advertisement of this program in local community centers, local schools, and health centers as this may increase the likelihood of women seeking and utilizing these resources. Many of the participants reported they had become aware of the Victims of Crime (VOC) program only when they were at a police station after they had filed a police report. Through the art of word of mouth, women would be able to offer support to each other and provide information about resources that might be available through local partnerships with the schools and community mental health centers and medical health centers. The use of the Spanish language media may be one of the most successful approaches because of the influence the media can have for this community.

The data gathered from participants in this study provided an understanding of the impact of violence on immigrant Latina women and their psychological well-being. It also provided a voice and visibility for immigrant Latina women who have historically lived in shadows of communities in the United States. Specifically, the study aimed to understand what motivated Latina immigrant women to seek mental health care; it aimed to identify obstacles that immigrant women encountered and prevented them from seeking mental health care; and finally, it sought to identify strategies on how current clinical practices may be tailored to address the mental health needs of this population.

While there are a number of evidenced-based practices such as prolonged exposure therapy, trauma-focused cognitive behavioral therapy, eye movement desensitization reprocessing and other modalities which have been demonstrated to be successful in managing mental health symptoms, this study shows that they do not address the unique deep-rooted cultural historical experiences immigrant Latina women have endured ([47,48], & [45,49]). The deep-rooted sociopolitical and cultural factors remained unaddressed and did not result in schematic cognitive changes for these immigrant Latina women.

Thus, rather than having practitioners tell the women what to do, it would be more effective to empower women through the act of listening to their stories. This would also help practitioners to fine tune their abilities to honor the lived realities of the women and their own individual psychology in social contexts. In doing so, immigrant Latina women would become empowered to break the cycle of oppressive patterns on their own terms. Also, practitioners could be mindful of working collaboratively to increase the level of consciousness through their own psychological awareness. This process would empower women to define their own lives rather than reinforce the systemic oppressive and demeaning nature of telling them what to do. The goal would be for women to increase the usage of mental health care and become empowered, breaking the cycle that dis-empowers women of color [40,50].

Not addressing the core roots of the problems will perpetuate the cyclical nature and maintain the status quo. Practitioners are in a position of power and can use their power by serving as advocates maintaining an active social justice role. In order to be effective and address immigrant women's psychological and emotional mental health, psychologists, social workers, counselors and other mental health providers should address the needs of the women based on their told stories, their realities. Not doing so will continue to reinforce the idea that those in power who have become experts in people's lives still do not work for the psychological well-being of the people.

Specific treatments such as trauma-focused cognitive behavioral therapy and eye movement desensitization reprocessing have been successful in the reduction of symptoms and treatment outcomes [51]. However, deep-rooted issues remain.

Feminist theory has offered interventions that uniquely address the needs of women and give a voice to women, and efforts have been made to establish equal political, cultural, social, and economic rights [52]. However, these have been done through a Western Eurocentric approach [52,53]. A critique of feminist theory referred to as *epistemic privilege* has argued that feminist philosophers have enjoyed a position of privilege not afforded to the living experiences of the majority of women in Latin America [45,[53], [54], [55], [56]].

Cognitive behavioral therapy has been known to be an effective approach to reduce anxiety and depression [57]. Cognitive behavioral therapy posits that identifying an individual's cognitive distortions or beliefs could result in improved behavioral interactions and improved mood. Although it briefly explores core schemas, it does not address the historical and cultural traumas immigrant Latina women have lived. Additionally, it shies away from long-term treatment interventions which often are needed to address these types of traumas and deeply ingrained schematic beliefs such as marianismo and fatalism in immigrant Latina women [57,58]. A heavy emphasis to challenge the status quo by validating their individual needs, reiterating their basic need for safety and security and viewing self beyond their family roles may be the most powerful intervention to empower minority subgroups.

### 4.3 Limitations

This study only included research on 20 immigrant Latina women who were between the ages of 27 and 58 years old and who had immigrated from their home countries to the United States. Subsequently, the women in this study had experienced interaction with receiving mental health care. This study did not include Latina women who were born in the United States and did not include women under the age of 18. Consequently, the experiences of these groups were not captured, and the data were limited to immigrant, adult Latina women. Additionally, the data collected were gathered utilizing a small convenience sample of immigrant Latina women living in the San Fernando Valley in Los Angeles County, California.

Furthermore, there were several limitations to the study in relation to the convenience sample used, including criteria related to women living in a specific geographic area who had had experience receiving mental health services. Retrospective experiential data was reported by the participants, some of whom had immigrated to the United States at an early age and others who had immigrated as adults. The level of acculturation of participants was a significant factor in the experiences these women endured based on the amount of time they had been in the United States. Language barriers which limited access and opportunities impacted the study; these languages included English, Spanish, and Quiché. Geographical and cultural differences from the various Latin America countries and between the participants were another factor in the study. Finally, the process of data translation from Spanish to English may have impacted the findings. The issue of acculturation was addressed by including participants who had lived in the United States for different lengths of time. Additionally, language barriers were addressed through the use of audio recording and verbatim transcription. Another limitation to this study was that the researcher was born in the United States from immigrant Latino parents from Mexico. The primary researcher was of the male gender which could have impacted the participants openness during the interviewing process.

## 5 Conclusion

The implications of this study for immigrant Latina women living in the United States reinforces the need for this type of research within these communities. This information may be relevant to other minority groups and other women of color who immigrated to the United States and experienced similar circumstances. Additionally, the findings in this research can serve as a starting point for others to conduct further research as well as highlight physical and mental health resiliency of immigrant Latina women. Latina women immigrating to the United States carry with them major psychological responsibilities and a burden on their psyche and mental health. Understanding barriers that may prevent immigrant Latina women from seeking and accessing mental health care can provide opportunities to improve the efforts to reach more women in the community. Taking into consideration motivating factors for immigrant Latina women to utilize mental health care is essential to building trust and the relationships between the women and the practitioners. Furthermore, practitioners have a responsibility to the professional practice of psychology to continuously work toward ensuring that people receive adequate mental health care to address their psychological and emotional well-being.

Finally, the issue of violence against women has been researched and studied over the years, and further work is needed to identify psychological treatments that may be the most effective for immigrant Latina women. The need for continued research on the topic of violence, women, and mental health care needs to be prioritized in the field and practice of psychology, not only in a micro-systemic level (i.e., therapy) but also at the macro-systemic level (i.e., outdated laws, patriarchal attitudes and belief changes). Thus, it is an indicator for increased need to address the unique psychological experiences of minority groups to include an understanding of the cultural and historical traumas and the violence experienced by these groups.

## Declaration of Competing Interest

The authors declare that they have no known competing financial interests or personal relationships that could have appeared to influence the work reported in this paper.
